# 3D Printing of Rapid, Low-Cost and Patient-Specific Models of Brain Vasculature for Use in Preoperative Planning in Clipping of Intracranial Aneurysms

**DOI:** 10.3390/jcm10061201

**Published:** 2021-03-13

**Authors:** Maciej Błaszczyk, Redwan Jabbar, Bartosz Szmyd, Maciej Radek

**Affiliations:** Department of Neurosurgery, Spine and Peripheral Nerves Surgery, Medical University of Łódź, 90-549 Łódź, Poland; rredwanbakal@gmail.com (R.J.); bartoszmyd@gmail.com (B.S.); maciej.radek@umed.lodz.pl (M.R.)

**Keywords:** 3D printing, aneurysmal clipping, rapid prototyping, surgical planning, neurosurgery

## Abstract

We developed a practical and cost-effective method of production of a 3D-printed model of the arterial Circle of Willis of patients treated because of an intracranial aneurysm. We present and explain the steps necessary to produce a 3D model from medical image data, and express the significant value such models have in patient-specific pre-operative planning as well as education. A Digital Imaging and Communications in Medicine (DICOM) viewer is used to create 3D visualization from a patient’s Computed Tomography Angiography (CTA) images. After generating the reconstruction, we manually remove the anatomical components that we wish to exclude from the print by utilizing tools provided with the imaging software. We then export this 3D reconstructions file into a Standard Triangulation Language (STL) file which is then run through a “Slicer” software to generate a G-code file for the printer. After the print is complete, the supports created during the printing process are removed manually. The 3D-printed models we created were of good accuracy and scale. The median production time used for the models described in this manuscript was 4.4 h (range: 3.9–4.5 h). Models were evaluated by neurosurgical teams at local hospital for quality and practicality for use in urgent and non-urgent care. We hope we have provided readers adequate insight into the equipment and software they would require to quickly produce their own accurate and cost-effective 3D models from CT angiography images. It has become quite clear to us that the cost-benefit ratio in the production of such a simplified model is worthwhile.

## 1. Introduction

Aneurysmal clipping belongs to one of the most difficult and demanding of neurosurgical procedures [1]. Complex anatomy, subarachnoid hemorrhage, often unpredictable intraoperative situation, and the risk involved with manipulation on a thin and delicate aneurysmal sac make the surgery of aneurysms challenging even among experienced professionals. Analysis of image studies such as Computed Tomography Angiography (CTA, CT-Angiography) or angiography plays a major role in preoperative planning. Thus far, the use of 3D-printing has gained interest among medical professionals, particularly in the personalized approach towards treatment. An example of such an approach in neurosurgery might be the use of 3D-printed brain vasculature of patients planned for aneurysm surgery. D’Urso et al. produced the first 3D model for complex intracranial aneurysms [2]. Wurm et al. further built upon this technique, creating three-dimensional models of cranium and complex cerebral vasculature [3]. Mashiko et al. created 3D hollow elastic aneurysm models and examined their utility for preoperative simulation [4,5]. Such models have found use in clip selection preoperatively, demonstrating the application of 3D printing in the modeling of intracranial aneurysms and its benefits preoperatively. Apart from the unquestionable benefits of the utilization of such models, the creation of them raises many technical issues. The most difficult to overcome is the adaptation of medical image files to use in 3D printing. Medical image data are stored as Digital Imaging and Communications in Medicine (DICOM) files—the standard for the communication and management of medical imaging information and related data [6]. DICOM standard has been developed by the American College of Radiology (ACR) and the National Electrical Manufacturers Association (NEMA) who formed the DICOM standards committee [7] to work on unifying the exchange and interpretation of medical image data and information related to them among professionals around the world. This cooperation allowed to introduce DICOM to every medical machine producing high-resolution digital images, becoming the default format in computed tomography (CT), magnetic resonance imaging (MRI), digital subtraction angiography (DSA), digital classic radiography, and every other imaging method utilizing the digital image acquisition.

Medical images are analyzed using DICOM viewers—special software usually provided by the manufacturers of imaging devices but also independent companies.

**Figure 1:**
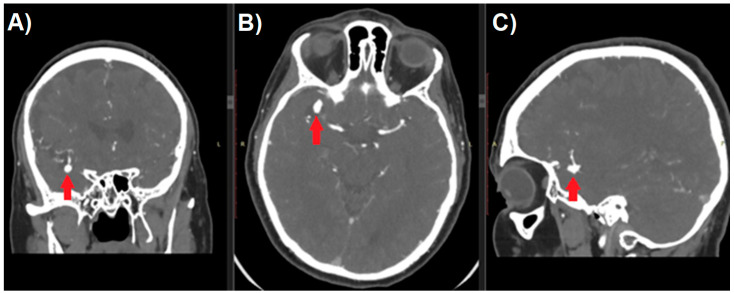
Multi-planar reconstruction of Computed Tomography Angiography study of patient with cerebral aneurysm (red arrow). Digital Imaging and Communications in Medicine (DICOM) image in (**A**) coronal, (**B**) axial, and (**C**) sagittal plane.

**Figure 2:**
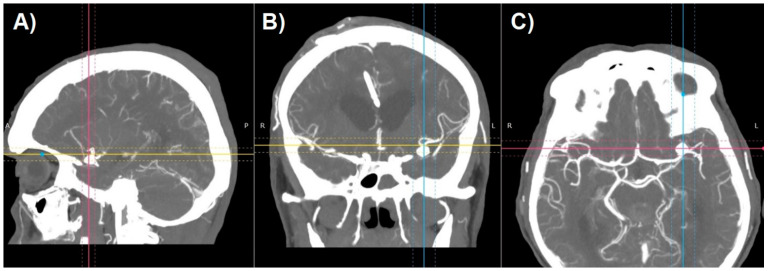
Maximum Intensity Projection reconstruction of CT-angiography of patient with cerebral aneurysm in (**A**) sagittal, (**B**) coronal, and (**C**) axial plane.

Aneurysm management starts with a thorough analysis of the anatomy. Raw image data from CT-angiography create axial slices through the examined part of the anatomy and can be converted to create sagittal and coronal projections (Figure 1). Unfortunately, because of the two-dimensional nature of such images, they usually fail to provide an accurate perception of treated pathology. Most DICOM viewers allow to perform the reconstructions of such images to create more like-3D images. Maximum Intensity Projection (MIP) and shaded surface display volume rendering techniques (SS-VRT) are mostly utilized. As it is shown in Figure 2, the first one consists of projecting several slices with the highest attenuation value on every view throughout the volume onto a 2D image [8]. Considering vascular diseases, this technique allows to trace the course of vessels neighboring to the aneurysm, thus producing the opportunity to visualize the pathology in a way accurate enough for preoperative planning.

**Figure 3:**
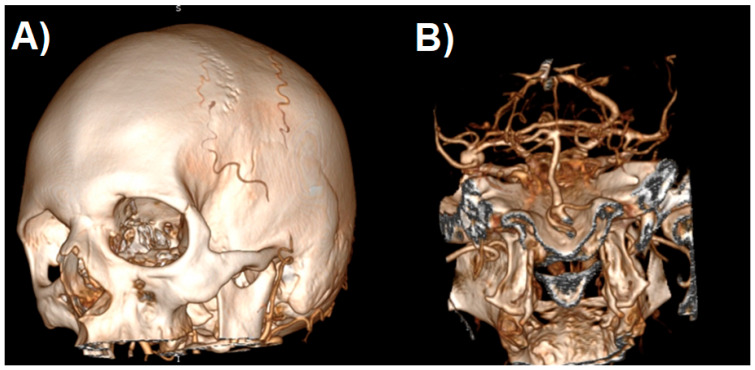
Shaded surface display volume rendering technique reconstruction of CT-angiography of patient with cerebral aneurysm: (**A**) raw reconstruction. (**B**) Circle of Willis after initial cropping.

SS-VRT is a technique facilitating the creation of a truly 3D visualization of CT volumetric data for display from any desired perspective (Figure 3) [9]. These techniques typically select voxels to be included in a surface rendering based on a selected range of Hounsfield values. By properly choosing the Hounsfield range, different types of tissues can be outlined like parenchyma, bone, vessels etc. Volume of CT data can be segmented into several of those tissue types according to the combination of Hounsfield ranges. These techniques then calculate the location of surfaces separating tissue types. The surface information is then used to calculate a perspective visualization based on observer position and light source positioning [10]. SS-VRT images provide a sensation of three-dimensionality that seems to be superior to any sort of 2D image, as the model created that way can be rotated, tilted, zoomed in and out, which creates the greatest opportunity to perceive the orientation of treated pathology. Both of aforementioned techniques, however helpful, remain only a two-dimensional presentation of three-dimensional content on the screen.

Creation of physical objects, depending on the technology involved, usually required technically complicated and time-consuming processes of creation of injection mold and their postprocessing or usage of CNC (computerized numerical control) milling machines. The necessity of involvement of expensive machinery park resulted in a situation where production of objects was reserved only for industrial purposes. Hope for bringing creation homes was the introduction of home-use desktop 3D printers. Among many different technologies of print, Fused Deposition Modeling (FDM) remains the most common form of 3D printing today. To form an object, the printer heats a cable of thermoplastic filament into liquid form and extrudes it layer by layer. Wide accessibility of cheap devices, free software, together with large support from 3D printing communities, ended the situation in which the production of objects was available uniquely in the industrial environment. The limitation of the DICOM system in the case of potential use for 3D printing is primarily the fact that it does not store information of a three-dimensional nature. Like JPG, TIFF, or GIF files, it presents graphics in the form of a map containing mostly a cross-section through all the components of the anatomy of the studied area, while the goal of the printout is rarely the complete system of organs.

To extract the data to be a digital prototype of a given 3D model, it is necessary to “clean” the initial DICOM study, which, if you want to model the vascular system, leaves only the vessels filled with contrast, e.g., the image obtained during the CT-Angiography examination or angiography. Three-dimensional reconstructions based on SS-VRT reconstruction, together with the increasing hardware capabilities of currently used computers, enabled the implementation to DICOM viewer tools for exporting 3D reconstructions to files containing spatial information (e.g., STL—Standard Triangulation Language), and only this type of file can be used as a material for 3D printing. Therefore, we aimed to assess the ability to create a 3D printout of the Circle of Willis model for use in the preoperative planning and to estimate the speed achievable at the moment, which would make the creation of a patient-specific model technically possible shortly before the surgery itself.

## 2. Materials and Methods

This is a prospective study of three patient-specific models of patients diagnosed with intracranial aneurysm admitted to the Department of Neurosurgery of Medical University of Lodz. The core data used for planning were CT-Angiography images and VRT reconstruction performed on its basis by RadiAnt^TM^ DICOM viewer (Medixant, Poznan, Poland).

An Original Prusa i3 MK3S^TM^ printer ((Prusa Research a.s., Praha, Czech Republic)) and dedicated PrusaSlicer^TM^ software version 2.2.0+ (Prusa Research a.s., Praha, Czech Republic) was used for printing and model preparation. The FDM (Fused Deposition Modeling) printer was chosen due to the lack of time and labor-intensive postprocessing required. The only thing to be done after the printer is finished with the model is to remove the supports mechanically. The PLA (polylactic acid) was chosen as a printing material due to desired stiffness, ease of printing the details, and lack of harmful fumes generated.

### 2.1. Preparation of STL File

**Figure 4:**
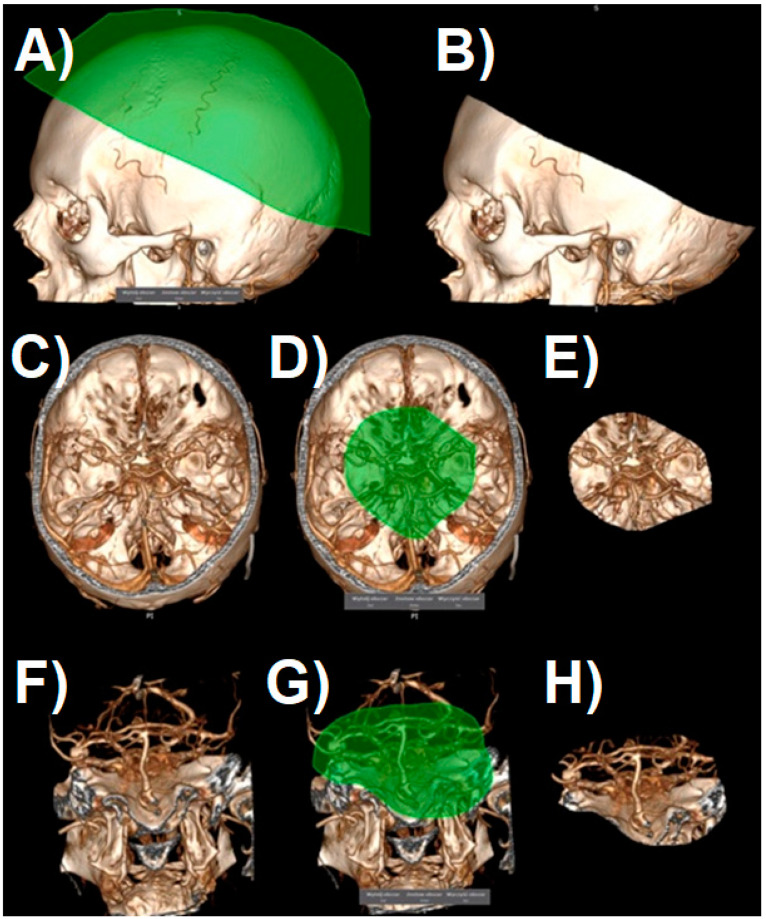
The process of cropping a model to the desired layout. Shaded surface display volume rendering technique reconstruction. The following images (**A**–**H**) represent the next steps.

After generating the VRT reconstruction in DICOM viewer, we manually removed the anatomy components that were not part of the future print using the “cut area” and “leave area” options. In practice, we removed most of the vaults and the base of the skull, leaving only the Arterial Circle of Willis with fragments of temporal and sphenoid bones (Figure 4). Such a model is usually sufficient to view the treated pathology from different angles.

**Figure 5:**
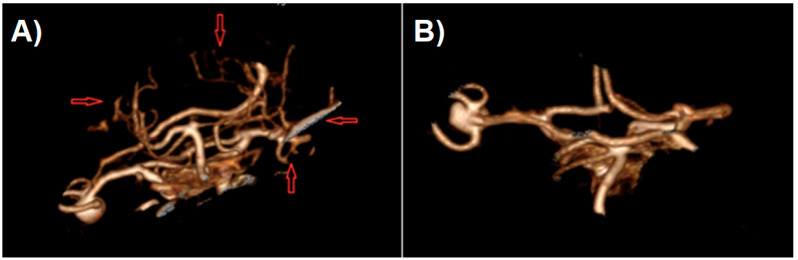
Artifact removal process: (**A**) before and (**B**) after.

In the next step, the digital 3D model should be cleaned off of unnecessary artifacts as otherwise they become part of the STL file, which would unnecessarily complicate the print. Artifacts can be smaller vessels, fragments of the skull, or anything that the algorithm creating the reconstruction has unnecessarily turned into a 3D model. Cleaning consists of two stages: in the first stage, we regulate the saturation to best visualize all the vessels we want to be part of the model. If for some reason we want to see smaller vessels that will appear at higher saturation, we should be prepared to deal with more artifacts. The second stage is to remove all SS-VRT visualization elements that we do not want to show on the created model. It is worth spending some time on these activities as they significantly affect the model quality (Figure 5).

The finished reconstruction is then exported to the STL file. Before the next stage, it is worth running the file in any 3D object viewer to assess the degree of “model cleaning” and repeat the last step if necessary.

The STL file already contains digital spatial information in the form of a polygon mesh. Regardless of the source of such a file, it must be converted into an execution algorithm for a 3D printer—the so-called G-code. Such a file will contain movement instructions for the printer head and metadata in the form of information about the temperature of the nozzle, table, head cooling parameters, and acceleration values for the printer’s stepper motors—it is therefore a compilation of information about the 3D model itself and hardware parameters. The latter will be different for different printers (even those using the same technology) or different materials.

The “Slicer” type software is used to generate the G-code file. It is usually delivered with the printer, but it is not necessary to use only the manufacturer’s software, e.g., when we want to edit some specific print parameters. Most popular slicers contain at least basic sets of settings for the most popular printer models.

### 2.2. G-Code File Preparation

**Figure 6:**
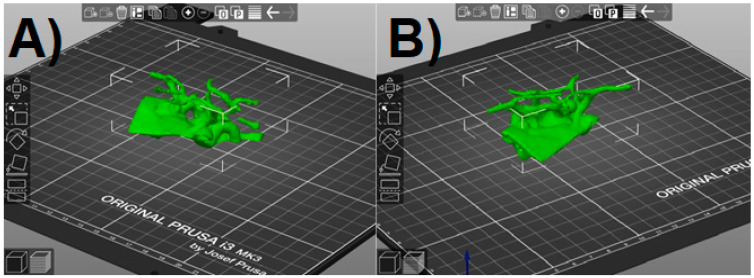
PrusaSlicer window with the Standard Triangulation Language (STL) model loaded. (**A**,**B**) view in different angles.

We ran the prepared STL file in the slicer. The program will randomly orientate it on the virtual printer table, which is then corrected using the available positioning tools (Figure 6). At this stage, we also have the option to resize the model to the desired value. It should be remembered that a small model will be more susceptible to damage when tearing off the supports, and a larger one will unnecessarily extend the printing time. The final size was selected arbitrarily so that it did not exceed approx. 10 cm at the widest point. As this software allows us to freely change the dimensions of the model on each axis, the scaling factor should be kept in order not to disturb the proportions.

**Figure 7:**
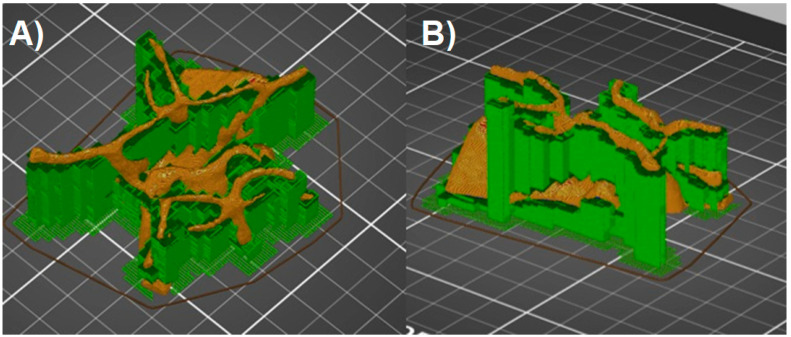
Model with supports added (green color). (**A**,**B**) view in different angles.

The FDM technology forces the model to be created in one plane, layer by layer. The extruded material is deposited on the printer table, and each subsequent layer is created on the previous one. This means that none of the model elements can theoretically be printed in the air. In practice, it is possible to print inclined planes when the so-called overhang threshold does not exceed the assumed angle, and the layers that form them lie directly on top of each other. Printing completely horizontal lines in the air is possible only when there are previously created points on the print, between which the filament is spread (Bridges). The shape of Willis’s Arterial Circle is therefore an example of a model whose parts are at least partially suspended above the printer table. Printing such a model is possible only when supports are used. These elements constitute a platform for the layers of the model, which would otherwise be suspended in the air and, practically during printing, would fall on the printer table in the form of filament threads, completely ruining the model. Supports can be printed from the same material as the actual print, or soluble materials, which significantly improves the quality of the printout, but requires a printer with several nozzles (PolyJet), or equipped with a filament change system during operation. Although possible in the case of the Prusa printer—associated with the need for its hardware expansion, longer printing time, and subsequent processing, and much greater material losses—the filament changes two or more times during each layer and the printer cleans the nozzle to ensure printing with a specific filament in given places. Most slicers can automatically add supports wherever the overhang threshold exceeds the allowable value (Prusa Slicer defaults to 55 degrees). From the point of view of Willis’ Arterial Circle printing, it is important to set the generation of supports everywhere (e.g., not just above the table itself), as some parts of the model hang over previously printed model elements. When using the automatic settings for generating supports, the default value of the distance in the Z-axis for the connecting layer needs to be changed to 0.2 mm (detachable supports)—this deteriorates the quality of the layer directly above the support, but the model will not be damaged during the attempt to tear the supports off. When using a soluble material for the supports, the Z-axis spacing can be left at the default value (0 mm). Finally, the program will “slice” the model with the supports, e.g., divide it into layers with a given thickness, and then save it as a G-code file, adding information about the print parameters for a specific type of filament (Figure 7).

### 2.3. Print and Post-Processing

The process of printing the Circle of Willis model itself does not differ in any way from printing other items. Calibration, cleaning, and the basics of using a 3D printer go beyond the scope of this manuscript. Invariably, for the FDM technology, a very important element is the careful observation of the formation of the first layer as the defect there is most likely to result in a failed print.

**Figure 8:**
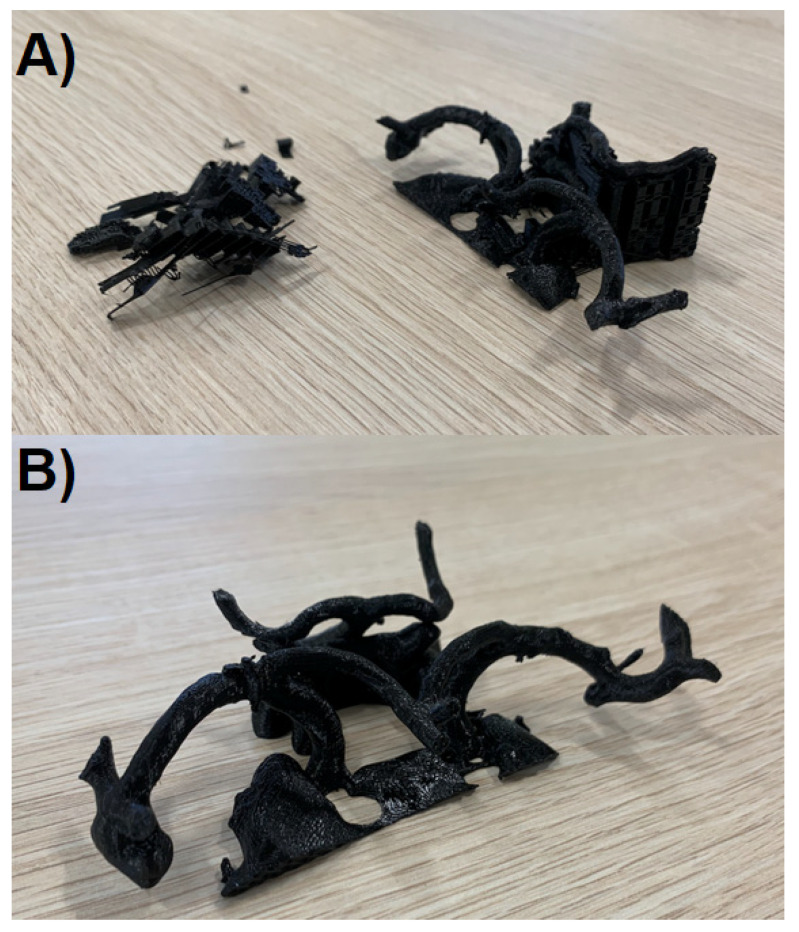
The obtained 3D models: (**A**) during the process of support removal, (**B**) final product.

The last element of model preparation, once the printer has completed its task, is the removal of supports (Figure 8A). In case the model is made entirely of one filament type, it should be done mechanically with pliers and prongs. Every effort should be made not to damage the model, because the elements of the supports may be strongly attached to the printed vessels in some places. The set of Non-Default Settings for Circle of Willis Model Printout (PrusaSlicer, Prusa Research a.s., Praha, Czech Republic) is shown in Table 1.

### 2.4. Evaluation of Obtained Models

Models were evaluated by the members of our neurosurgical team (*n* = 10) for quality and practicality. Doctors were asked for (1) their assessment of the quality of anatomical structure visualization using classical radiological approaches and proposed 3D models; (2) their median comfort while performing aneurysm clipping procedures with and without the aid of 3D models; (3) their perceived median level of patient safety during clipping procedures aided by classical radiological approaches and proposed 3D models. Moreover, they were surveyed for (4) their perceived median difficulty level of 3D model preparation, (5) their willingness to use the resultant models, (6) their usefulness in clinical practice, as well as (7) the cost affordability. All answers were given on a 0–10 scale.

### 2.5. Statistical Analysis

Surveyed data were reported as a median (1. quartile—3. quartile). The dependencies in the assessment of the quality of anatomical structure visualization, comfort during surgery, and the perceived median level of patient safety during procedures performed with and without 3D models were tested using the Wilcoxon test. A level of 5% was used as a significance threshold for all the results. Statistical analysis was performed using STATISTICA 13.1 (TIBCO, Palo Alto, Santa Clara, CA, USA).

## 3. Results

We successfully proved the possibility to produce 3D-printed models showing not only aneurysms and the parent vessels but the entire Circle of Willis in the simplest way possible utilizing an affordable 3D printer and free software. The median time needed for us to create the model illustrated in Figure 8B was 4.4 h (range: 3.9–4.5 h). This short duration of time therefore allows us to deploy such models in preoperative planning for both emergency and non-emergency scenarios. The overall feedback we received from surgical teams and educators that used our models were positive. As our models were scaled versions of real anatomical structures and pathology, operative teams found them to be particularly useful in selecting the equipment they would require, such as clips. In many instances, we have also found our models to be repurposed for educational needs: for example, in obtaining informed consent. We therefore believe our 3D-printed models bear much value for little cost and effort required to produce them. The printer used is priced 769 €–1000 € according to the variant chosen which, in comparison to the cost of equipment necessary for surgical treatment, has a chance to be relatively negligible in the hospital budget.

### Evaluation of Obtained Models

**Figure 9:**
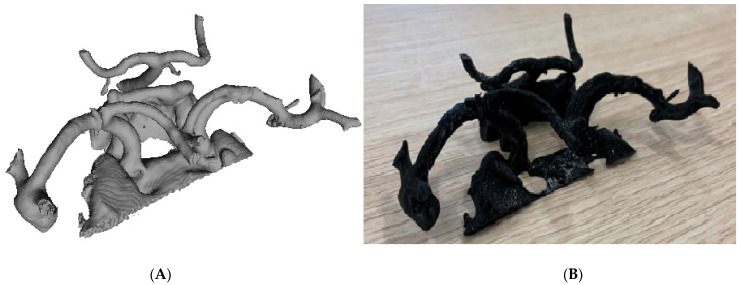
(**A**) STL file derived from CT-Angiography; (**B**) final 3D-model.

Performed statistical analysis revealed that use of aneurysm 3D-printing ameliorates: (1) the quality of anatomical structure visualization before surgery from 8 (IQR: 7–8.75) to 10 (IQR: 9.25–10) points (*p* = 0.008; see Figure 9); (2) doctors’ comfort from 8 (IQR: 6.25–8) to 9 (IQR: 8.25–10) points (*p* = 0.012). Moreover, the use of 3D models reduced the median level of concern for patient safety from 8.5 (IQR: 8–9.75) to 6 (IQR: 5–7.75) points (*p* = 0.008).

The median model preparation difficulty level was 3.5 (IQR: 2.25–4.75). The neurosurgeons surveyed find these 3D models useful and affordable in routine practice (10 (IQR: 10–10) and 10 (IQR: 8.5–10), respectively). Thus, they would like to continue to use them in their practice: 10 (IQR: 10–10).

## 4. Discussion

Literature on the use of 3D-printed models as a prompt prototyping technique is on the rise, predominantly due to the increased accessibility, ease of use, and affordability of 3D printers [11,12,13,14]. Many publications indicate the potential application of 3D printing in the modeling of intracranial aneurysms and its benefits preoperatively [3,4,5,11,15,16,17]. Invitro models of the biological effect of complex-flow stress on endothelial lining, hemodynamic aneurysm growth, aneurysm rupture, and treatments are just a few of the many instances in which researchers relied on 3D-printed models to both present and educate others on their research findings [18,19,20,21,22]. The use of 3D-printed models has also been greatly successful and found to be an excellent tool in simulations, specialized training as well as patient education [18,23,24].

Although literature is rich in papers presenting possible applications of 3D print in neurosurgery, it lacks articles detailing manufacturing steps of developing personalized rapid 3D prints. This article discusses a simplified and fast method to produce individualized 3D printouts that are geometrically and functionally accurate.

The total amount of time used for the creation of a model was about 4 h on average—from CTA acquisition to the completed model. Faraj et al. note that it would take them between 24 to 28 h on average to produce a model. Kimura et al. express in their publication that it would take them 3–7 days. Wurm et al. and Chueh et al. both disclosed that they required 1.5 weeks and 92 h, respectively [3,17]. Our methodology appears to be more streamlined, applying the standard method outlined in our article, to manufacture models rapidly. This allows for rapid deployment of the model for surgical teams to be used as a tool to plan cerebral aneurysm clipping even in some emergency cases.

The assessment method we used for our 3D-printed models was visual, similar to other numerous studies where the authors reported the intraoperative anatomical relevance of their 3D-printed models with the reference to visual comparison to the dimension and configuration details illustrated by the models [20,25,26]. Nonetheless, the recent trend is leaning toward the more objective evaluation and observation, such as statistical analysis of aneurysm diameter, length, and thickness measurement relative to radiographic image—computational comparison of re-imaged model—and DSA images—visual comparison of model and CTA and DSA to intraoperative anatomy [4,27,28,29,30,31]. In all these studies, there was not any significant difference between preoperative imaging and printed models, suggesting that these printed models are anatomically accurate [3,4,29,31]. In this study, only the location and orientation of aneurysm corresponding to the parent vessels were assessed, and we found an accurate reflection of intraoperative anatomy, with no further stress on the surrounding structures and small perforators [15,31].

The material our team used to create the models was PLA as it was mandated by the 3D printer. Given the material we used, the consistency of the models was significantly more rigid than the real anatomical vessels. Thus, our models were not suitable to be used to practice vessel dissection and clipping. While the utilization of other types of filaments can produce a more flexible and realistic aneurysm, that would serve better in preoperative simulation and training scenarios, the deployment of such a model in emergency situations does not seem feasible at this point. Examples of such endeavors were undertaken by Kang, Wurm, and Mashiko et al. Kang’s team produced flexible 3D models of an aneurysm in a rigid skull [32]. Wurm and Mashiko et al. both presented variants of hollow models, which permit assessment as well as the testing and positioning of different clips [4,16]. Despite that the models we created are lacking such properties, the cost and the time necessary to create a similar one would be significantly greater, and our team’s goal was to quickly produce models, at an affordable cost, for rapid deployment to surgical teams.

## 5. Conclusions

Technologies once available only to industry are gradually becoming available and affordable within the consumer market. Medical imaging industries have begun to offer services that convert patient-specific geometrics, taken from a CT or MRI, directly into STL files using specific software, making the process of rapid 3D printing far easier than it has been historically [33,34,35,36]. The process of creating models of cerebral vascularization, in patients treated for intracranial aneurysms, although complex and requiring the use of special software and hardware, is still simple enough to allow any person familiar with the process of 3D printing to create a useful model in hospital settings. As studies have yet to be published detailing the manufacturing steps of developing personalized rapid 3D prints, we hope that we have successfully provided you with some insight into how these models are produced as well as their practicality and usefulness in fields such as vascular neurosurgery.

## Figures and Tables

**Table 1:** Set of Non-Default Settings for Circle of Willis Model Printout (PrusaSlicer).

Parameter	Value
Layer thickness	0.15 mm (Quality)
Material	Generic PLA
Supports	Everywhere
Contact Z distance	0.2 mm—detachable supports

## Data Availability

Data are available upon request, please contact Maciej Błaszczyk (maciej.blaszczyk@umed.lodz.pl).
